# How Does Extended Maceration Affect Tannin and Color of Red Wines from Cold-Hardy Grape Cultivars?

**DOI:** 10.3390/foods14071187

**Published:** 2025-03-28

**Authors:** Aude A. Watrelot, Nicolas Delchier

**Affiliations:** Department of Food Science and Human Nutrition, Iowa State University, 536 Farm House Lane, Ames, IA 50011-1054, USA; delchier@iastate.edu

**Keywords:** phenolic compounds, tannins, anthocyanins, Marquette, Petite Pearl

## Abstract

Red wines produced with interspecific grape cultivars tend to have low tannin concentration and are therefore unbalanced. Extended maceration (EM) is a common winemaking technique which can promote the extraction of tannins from grape skins and seeds. The goal of this study was to evaluate the effect of EM on the tannin concentration, color intensity and other chemical properties of red wines made from cold-hardy grape cultivars. The wines were made from two cold-hardy interspecific grape cultivars (Marquette, and Petite Pearl) for either 7 days (control) or 21 days (EM) before pressing. Chemical analysis of the wines was conducted to determine their tannin concentration and color parameters at different stages of the process and after 14 months of aging. EM resulted in an improvement in the iron-reactive phenolic content of Marquette red wines (from 582 to 969 mg/L at bottling in control and EM wines, respectively), but no significant improvement in tannin content. The hue of Petite Pearl wines increased following EM only at pressing, and color intensity of those wines decreased at pressing and bottling by 43% and 52%, respectively. This study was the first one conducted on non-*Vitis vinifera* grapes which showed a lack of impact of EM on the phenolics and tannin concentration in the red wines made in 2022.

## 1. Introduction

Interspecific cold-hardy grape cultivars have been developed for their ability to survive to harsh cold climate, as well as help improve the chemical and sensory attributes of Native American grapes [1]. However, the wines produced from these cold-hardy grapes tend to have a low tannin concentration, and a high acidity leading to a lower quality [2,3,4].

Tannins and anthocyanins are the two main phenolic compounds in red wines responsible for astringency, color stability, and protection against oxidation, and therefore its high quality [5]. Condensed tannins, also called proanthocyanidins, are located in grape skins, seeds and stems. They are flavonoids composed by subunits including (-)-epicatechin and (-)-epigallocatechin mainly in skins and epicatechin-3-O-gallate in seeds, with the number of constitutive units corresponding to the mean Degree of Polymerization (mDP), that is higher in grape skins than in seeds [6]. Also, a high mDP induces a stronger binding affinity with proteins and cell wall material that limits their extraction from skins or seeds. As a result of their structure and size, tannins are not aqueous-soluble and can be extracted only after several days of alcoholic fermentation. In addition, anthocyanins are the red pigments found in grape skins and sometimes in grape flesh. The most common anthocyanin found in grapes is malvidin-3-O-glucoside which provides a purple red color at low pH. However, in interspecific grape cultivars, anthocyanin concentrations tend to be higher than in *V. vinifera* and are more abundant in diglucoside forms. Those pigments are water-soluble and are extracted from skins and flesh during the first days of the alcoholic fermentation [7].

Several winemaking techniques have been applied on interspecific grape cultivars to increase the final concentrations of tannins in red wines. For instance, Accentuated Cut Edges is a mechanical technique of cutting grape skins into small fragments, facilitating the extraction of phenolic compounds [8]. Enzymes have been used in addition to this technique in order to disrupt the cell wall material binding with phenolic compounds [9,10]. Temperature is a key factor to manage in order to increase the extraction of phenolic compounds. Cold soaking for a couple of days increases the extraction of anthocyanins while high-temperature treatment such as thermovinification increases the extraction of tannins by modifying the solubility and the porosity of the cell wall material of skins and seeds [11,12]. Moreover, studies have examined cap management techniques during fermentation to achieve faster extraction of phenolic compounds in red wines [13,14]. Punch-down, pump-over and submerged cap methods have been previously compared. A previous study investigated the combination effect of cap management and temperature on Pinot noir wines. Results indicated that higher fermentation temperature increased tannin concentration, while no punch down increased anthocyanin concentration [13]. However, no combined effect was observed on the basic chemical parameters, showing that fermentation temperature was the key factor for the phenolics extraction in Pinot noir wines. In addition to those techniques, extended maceration (EM) has been used since the beginning of winemaking technologies to increase phenolics extraction from grape skins and seeds, to both improve the color stability, reduce the risks of oxidation, and therefore to enhance the quality of wine during aging [15].

EM refers to the time that musts are left in contact with wine after alcoholic fermentation. Specifically, this time can be extended from a few days to months after fermentation to increase tannin extraction and their polymerization with anthocyanins leading to color stability [16]. Polysaccharides, from grapes and yeast cell walls, are also extracted more effectively with EM time [17]. Those macromolecules can therefore interact during EM, as a result of a higher solubility with the increase in alcohol concentration, and the degradation of skins and seeds. Moreover, EM leads to a decrease in anthocyanin concentrations in wines as they bind with either other anthocyanins or flavanols or tannins to form polymeric pigments, which are responsible for stable color [18]. As previously observed, tannin concentrations in Merlot wines after 10 days of fermentation was lower than after 30 days of EM. Also, the concentration of tannins after EM reached a plateau at the end of the maceration, likely due to desorption mechanisms of interactions between cell wall material and tannin that occur with ethanol [19,20]. In contrast, anthocyanin concentration was lower in EM wines than in control wines, due to the formation of polymeric pigments that occur between anthocyanins, oxygen, acetaldehyde and tannins. Frost et al., (2018) [14] evaluated the astringency perception (related to tannin concentration) of Merlot wines when subjected to either no EM or up to 8 weeks of EM. Astringency perception was higher for wines subjected to 6 weeks of EM than for control wines and those with different EM durations. Even though EM was supposed to be a very efficient technique to improve tannin extraction and the overall wine quality, there is still a lack of experimental rigor and data on the impact of this technique on red wine quality, especially for interspecific cold-hardy grapes.

In this study, we hypothesized that interspecific cold-hardy grape wines that are poor in tannins and rich in anthocyanins would benefit from a 14-day EM to both enhance the tannin extraction and the polymeric pigment formation, to obtain a stable color.

## 2. Materials and Methods

Chemicals

Sodium hydroxide, hydrochloric acid, ferric chloride, *ortho*-phosphoric acid (85%), acetonitrile (HPLC grade), ammonium dihydrogen phosphate (98–99%), and potassium metabisulfite (KMBS) were provided by Fischer Scientific (Fair Lawn, NJ, USA). Sulfur dioxide (SO_2_) reactant, SO_2_ titrant, and SO_2_ acid solution were provided by Vinmetrica (Carslbad, CA, USA).

Winemaking

Interspecific grape cultivars Marquette and Petite Pearl were grown on a single high-wire trellis system at the Iowa State University (ISU) Horticulture Research Station (Latitude 42.10727, Longitude −93,585,981). Marquette grapevines were planted in 2011, and Petite Pearl grapevines were planted in 2019. The grapes were grown in moderately eroded Clarion loam soil. Marquette grapes (54 kg) were manually harvested on 26 August 2022, and Petite Pearl (54 kg) grapes were manually harvested on 9 September 2022. Grape clusters were sprayed with an SO_2_ solution (30 mg/L) before being transferred to the ISU winery for processing. As previously described [21], grapes were crushed and destemmed using a crusher/destemmer (Zambelli Enotech, Camisano Vicentino, Italy), and 6.5 kg of must from each cultivar was placed in an 8 L bucket, in triplicate. All the conditions of control and EM went through the same process of alcoholic and EM. Alcoholic fermentation was performed on the same day, by inoculating Lalvin ICV D254 yeast strain (Scott Laboratories, Petaluma, CA, USA) at 0.25 g/L, and GoFerm^TM^ yeast rehydration nutrient (Scott Laboratories, Petaluma, CA, USA) at 0.3 g/L following manufacturer’s instructions. After 48 h, malolactic fermentation was started by co-inoculating Lalvin VP41 bacteria (Scott Laboratories, Petaluma, CA, USA) at 0.01 g/L following manufacturer’s instructions.

Punch down was performed twice daily. Temperature and degree Brix were monitored daily following the second punch down, using a portable density meter (DMA 35, Anton Paar, Ashland, VA, USA), and are reported in Figure S1.

Control wines were pressed into 4 L glass jugs, using a benchtop press, after the completion of the alcoholic fermentation (6 days). These jugs were closed with an airlock, filled with a SO_2_ solution, until completion of the malolactic fermentation. Then, wines underwent cold stabilization at 6 °C for 1 month. The EM treatment group was maintained at room temperature (20 °C) for an additional 14 days. The EM treatment buckets were flushed with nitrogen every week to reduce the risks of oxidation and microbial spoilage and were closed with a lid mounted with an airlock filled with a SO_2_ solution. After 7 days of EM, lids were opened to assess the quality, and one punch down was performed to mix the wine. Then, 2.4 mL of a 6.6% SO_2_ solution was added to each bucket before flushing with nitrogen gas and sealing with the lids. EM continued for an additional 7 days. Wines were finally pressed into 4 L jugs using the same benchtop press, flushed with nitrogen gas, and stored at 6 °C for 1 month. Control wines and EM wines were carried out in triplicate for both Marquette and Petite Pearl cultivars. After cold stabilization, control and EM wines were bottled into 375 mL amber bottles with 30 mg/L free SO_2_, flushed with argon and closed with #9 conglomerated corks. Wines were stored horizontally at 15 °C for 14 months.

Chemical parameter measurement

Chemical parameters were analyzed in all samples at crushing, pressing, and bottling and after 14 months of aging. The pH was measured using an Orion Star ^TM^ A211 Benchtop pH meter (Thermo Fisher Scientific, Waltham, MA, USA). Titratable acidity (TA—expressed in tartaric acid equivalent) was measured on a 5 mL must or wine sample, by titration with sodium hydroxide (0.1 N) to an endpoint pH of 8.2. Degree Brix of juices at crushing was measured with a digital refractometer RF153 (FLIR commercial systems Inc., Nashua, NH, USA).

All samples were centrifuged (AccuSpinTM Micro 17 Centrifuge, Thermo Fisher Scientific, Waltham, MA, USA) at 16,200× *g* for 5 min prior to organic acids, ethanol quantification, as well as for color parameter determination.

Organic acids and ethanol were quantified using a high-performance liquid chromatography (HPLC) system (1260 infinity II, Agilent Technologies, Santa Clara, CA, USA) with a diode array detector (DAD) and refractive index detector (RID), as previously published by [8].

The color intensity and hue were calculated from absorbance values determined at wavelengths of 420 nm, 520 nm, and 620 nm, using a 1 mm quartz cuvette with a Genesys 150 UV-Vis Spectrophotometer (Thermo Fisher Scientific, Waltham, MA, USA). CIELab color coordinates were determined using 1 cm path-length UV–Visible cuvettes with a UV–Visible Spectrophotometer (Genesys 150, Thermofisher scientific, Waltham, MA, USA). The CIELab parameters including L* (lightness), a* (green/red component), and b* (blue/yellow component) were calculated using Visionlite software version 2.0 (Thermofisher scientific, Waltham, MA, USA).

Total Iron-Reactive Phenolics, SPP, and Anthocyanin Concentrations

The total iron-reactive phenolics (IRP) concentrations were evaluated using the Harbertson–Adams assay [22]. Wine samples were centrifuged at 16,200× *g* for 5 min (AccuSpinTM Micro 17 Centrifuge, Thermo Fisher Scientific, Waltham, MA, USA), and 75 μL of the supernatant was added to 800 μL of a buffer of sodium dodecyl sulfate/triethanolamine. The absorbance was measured at 510 nm and recorded after a 10 min incubation period, against a blank of 875 μL of a buffer of SDS/TEA. Then, 125 μL of ferric chloride reagent was added, and the absorbance at 510 nm was recorded after a 10 min incubation period. Total iron-reactive phenolics concentrations were expressed in mg/L as (+)-catechin equivalent.

The same “Harbertson–Adams” assay was used to determine the concentration of polymeric pigments (PPs) in wines [22,23]. Small Polymeric Pigments (SPPs) were expressed as absorbance units.

Anthocyanin concentrations were also determined following the “Harbertson–Adams” assay procedure using 100 μL of wine with 400 μL of model wine and 1 mL of anthocyanin buffer composed of maleic acid (23 g/L) and sodium chloride (9.93 g/L) with a pH of 1.8. After vortexing and 5 min of incubation, the absorbance at 520 nm was recorded and the concentrations were expressed as equivalent of malvidin-3-O-glucoside (M3G).

Tannin Concentration

Tannin concentration was quantified using RP-HPLC–DAD (1260 Infinity II, Agilent Technologies, Santa Clara, CA, USA) with a polystyrene divinylbenzene column (PLRP-S, 2.1 × 50 mm, 100 Å, 3 µm, Agilent Technologies, Santa Clara, CA, USA) and a guard column (PRP-1, 3 × 8 mm, Hamilton Co., Reno, NV, USA). Detection was recorded at 280 nm. Mobile phases consisted of 1.5% (*w*/*w*) 85% ortho-phosphoric acid (mobile phase A) and 20% (*v*/*v*) mobile phase A in acetonitrile (mobile phase B), with a flow rate of 0.30 mL/min. A linear gradient was started with 14% B, linearly increased to 34% in 12.6 min and held for 0.7 min, linearly increased to 70% in 1.8 min, held for 1.7 min, then decreased in 2.8 min and held at 14% B for 8.4 min. The column oven was set at 30 °C, and 5 µL of samples was injected after filtration through a 0.45 µm PTFE filter [24].

Statistical Analysis

Statistical analyses were performed, using JMP 16.0 software (SAS Institute Inc., Cary, NC, USA). Student’s *t*-test was used to compare the average of the different parameters (Brix, pH, TA, organic acids, color parameters, phenolics, tannins, anthocyanins and SPP) between wines made in triplicate with Marquette and wines made with Petite Pearl cultivars at crushing, with significance set at a *p*-value lower than 0.05. Two-way ANOVA was used to assess the impact of both time point (pressing, bottling and after 14 months of storage) and the treatment (control versus EM) for wines made from Marquette and Petite Pearl cultivars, with biological replicates (3 independent fermentation). For ANOVAs, significant differences were determined using a post hoc Tukey HSD test, with a 0.05 significance level (*p* < 0.05); *p*-values associated with the treatment, time point and the interaction between treatment and time point are presented in Supplementary Table S1.

## 3. Results and Discussion

### 3.1. Basic Chemical Parameters

Marquette grapes were harvested about two weeks earlier than Petite Pearl grapes with a higher degree Brix, higher TA and lower pH (Table 1).

The pH of Petite Pearl was at 3.15, while the pH of Marquette grapes was at 2.99. The TA of Marquette was 13.55 g/L, which is in agreement with other chemical parameters determined for Marquette grapes at crushing [2]. Concentrations of tartaric acid and malic acid in Marquette juices at crushing were about the same at 8 g/L, whereas Petite Pearl contained at least two times more tartaric acid than malic acid. The high concentration of malic acid in Marquette grapes has been shown to be a characteristic of *Vitis riparia* genetic heritage [25]. The hue was not statistically different between cultivars, but the color intensity was the highest in Petite Pearl juices. The L* a* b* color parameters showed that Marquette juice was orange–red compared to Petite Pearl juices which was deep red. The b* parameter, corresponding to the yellow (+b)—blue (−b) coordinate, was the only parameter that was significantly lower in Petite Pearl juice compared to Marquette juice. The concentration of phenolics was the same in both Marquette and Petite Pearl juices around 300 mg/L, but were much lower than the ones previously reported (1.112 mg/L in Marquette juice and 1.252 mg/L in Petite Pearl juice [26]). These differences might be due to the different method of analysis of phenolics used as the Folin–Ciocalteu method tends to overestimate phenolics concentration by considering all antioxidant compounds present in a plant sample [27]. The differences might also be due to the growing season, especially as this study has been completed on grapes only in 2022, and the location, as previously observed on Marquette grapes from Minnesota vs. Iowa, vs. Wisconsin [21,26]. As previously noticed [3,28], tannin concentrations were very low, less than 22 mg/L, indicating that no tannin was present in the juices. This was expected as condensed tannins from skins and seeds are extracted during alcoholic fermentation, resulting in a lower concentration in the juice. In contrast, anthocyanins are highly soluble in aqueous solutions, leading to their higher concentration in juices [29]. Anthocyanin concentrations were five times higher in Marquette juices (542.2 mg/L) than in Petite Pearl juices (109.9 mg/L), which was different to what was previously observed [26]. As suggested above, the differences between the previous research and this study might be the result of different locations and growing seasons.

During the winemaking process, the pH varied from 3.20 to 3.39 in Marquette wines, and from 3.18 to 3.33 in Petite Pearl wines (Table 2). TA varied from 7.91 to 12.58 g/L in Marquette wines, and from 7.35 to 9.57 g/L in Petite Pearl wines. As expected, the pH increased during the winemaking process while the TA decreased. However, no statistical impact of EM was observed on the pH and the TA for the different wines. Except for control wines made with Marquette, EM did not statistically affect the content of tartaric acid in the wines. Ethanol concentration was lower in Petite Pearl wines than in Marquette wines, as a result of a lower degree Brix in the grapes. The ethanol concentration varied from 8.62% to 12.04% in the wines and was not statistically impacted by the EM treatment. These concentrations were low, which is further discussed below, as the extraction of phenolic compounds, especially tannins, is impacted by the ethanol concentration of wines.

Malic acid concentration was statistically higher at pressing in Marquette control wines and Petite Pearl control wines compared to EM wines. The concentration of malic acid in the control wines was checked before pressing to identify when malolactic fermentation was complete. The concentration of malic acid was below the limit of quantification before pressing; however, the process of pressing must have released juice and malic acid from intact berries. This would explain the high concentration of malic acid at pressing in control wines. Lactic acid varied from 0.08 g/L (Marquette control pressing) to 1.63 g/L (Marquette control after 14 months). EM did not statistically increase lactic acid concentrations, except at pressing for both Marquette and Petite Pearl wines, showing that EM facilitated the completion of the malolactic fermentation. In control wines of Marquette and Petite Pearl, those higher concentrations of malic acid and lower concentrations of lactic acid suggested that malolactic fermentation was not completed at pressing. Acetic acid concentration varied from 0.29 g/L (Petite Pearl control at pressing) to 0.54 g/L (Marquette EM after 14 months). Acetic acid concentrations were statistically higher in Marquette wines made with EM for the three sampling points, but were below sensory thresholds of red wine (0.70 g/L). These results suggested that either some oxidation reactions started to occur or *Acetobacter* bacteria developed with time, even though the wines were stabilized by flushing with inert gas or with addition of sulfur dioxide during the time of maceration. In Petite Pearl wines, acetic acid concentration was statistically higher after EM only at pressing and after 14 months of storage.

### 3.2. Phenolics and Tannins Concentrations

**Figure 1:**
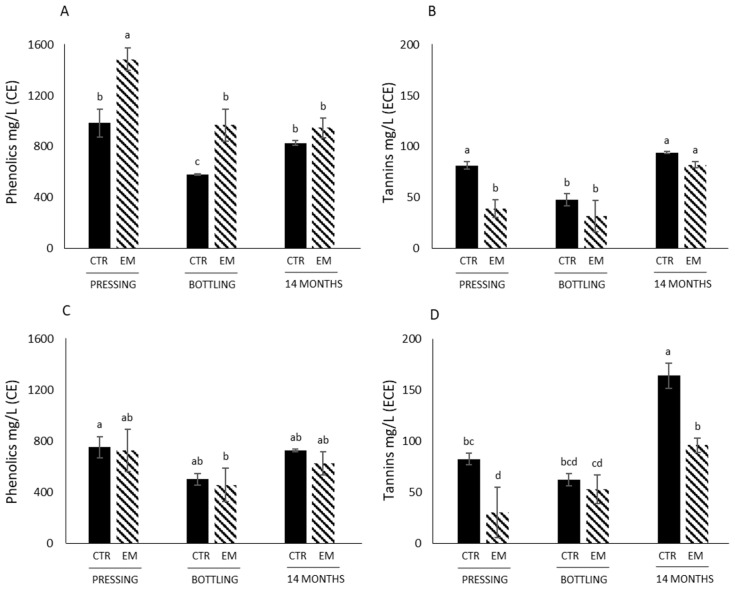
Phenolic and tannin concentrations in control and EM wines made from Marquette and Petite Pearl cultivars. (**A**) refers to phenolics concentration in wines made with Marquette cultivar; (**B**) refers to tannin concentration in wines made with Marquette cultivar; (**C**) refers to phenolics concentration in wines made with Petite Pearl; (**D**) refers to tannin concentration in wines made with Petite Pearl cultivar. Different letters indicate statistically significant differences (*p* < 0.05) by two-way ANOVA (time point versus treatment) followed by Tukey’s test.

Iron-reactive phenolics concentrations in Marquette wines varied from 581 mg/L CE eq. (bottling control) to 1490 mg/L CE eq. (pressing EM) (Figure 1A). Phenolics concentration decreased in Marquette wines from pressing to bottling, and then increased from bottling to 14 months of aging. Phenolics concentration in Marquette wines was significantly higher after EM at both pressing and bottling but showed no statistical differences after 14 months of aging (Figure 1A). In Petite Pearl wines, phenolics concentrations varied from 457 mg/L CE eq. (bottling EM) to 752 mg/L CE eq. (Pressing control) without any significant differences between treatments (control vs. EM) for all sampling time points (pressing, bottling, 14 months of storage) (Figure 1C). EM did not impact the content of phenolic compounds in Petite Pearl wines. Those results were somewhat contradictory to previous studies on Merlot and Cabernet sauvignon wines, which found a positive correlation between the duration of the maceration and phenolics extraction [14,19]. This has only been observed for Marquette wines at pressing and bottling (Figure 1A), suggesting that the cell wall material of both the grapes and yeasts were binding less with phenolic compounds, leading to a higher concentration of soluble non-precipitable phenolics. The results presented in this study were obtained in 2022 and should be taken with caution as the growing season and environmental factors impact the concentration of phenolic compounds in grape skins and seeds as previously observed [3]. However, although the results were obtained only for one growing season, the concentrations of phenolics and tannins in grape skins and seeds are always much lower than in a *Vitis vinifera* grape cultivar such as Pinot noir, suggesting that the current results might be similar during another growing season.

Tannin concentrations varied from 19 to 164 mg/L ECE eq., which was in agreement with concentrations of tannins observed in Marquette and Frontenac wines [21], but were very low compared to *Vitis vinifera* grape varieties such as Pinot noir or Cabernet sauvignon [30]. This seemed to be due to the low content of tannins originally found in the grape skins and seeds at harvest [3]. Concentrations were the highest in Petite Pearl wines after 14 months of aging (Figure 1D). Tannin concentration in Marquette wines decreased from pressing to bottling and then increased after 14 months of aging (Figure 1B). This decrease after pressing might be due to the tannin precipitation that can occur during cold stabilization, as previously observed [31]. However, this decrease was only observed in Marquette wines. EM did not statistically affect tannin concentrations in Marquette wines at the three different sampling points (Figure 1B). Similarly, EM exhibited significantly lower concentrations at pressing and after 14 months of storage for Petite Pearl wines, while concentrations were the same at bottling (Figure 1D). These results were contradictory to previously observed effects of EM, showing an increase in tannin concentration in wines after EM [14,19,32,33]. In addition, a recent study evaluated the impact of both the duration of maceration and the grape variety on the phenolics, tannins and sensory profile of two *Vitis vinifera* grape cultivars [15]. The authors showed a positive correlation between phenolics and tannins concentrations in wines and the length of maceration, with the highest concentration observed after 10 days of maceration. In another study, comparing the effect of extended maceration for 1 month and 6 months on red wine chemical properties of Zinfandel and Pinot noir cultivars [34], the concentration of tannins in those finished wines were not improved after 1 month of extended maceration, and was higher in Pinot noir wines after 6 months of extended maceration compared to Zinfandel. In all these previous studies, it has been suggested that the chemical composition of grape cultivar has an impact on the extraction of tannins, as well as on the desorption phenomenon of tannins during the short time of extended maceration. Tannin concentrations in finished wines is defined as the concentration of enological tannins added during the winemaking process (if any), and the tannins that are extracted from grapes, that did not adsorb, precipitate, and interact with other compounds from grapes and yeasts [35]. Tannins are soluble in alcoholic solution, and a higher concentration of ethanol facilitates their extraction in wine. In the Marquette and Petite Pearl wines, the concentration of ethanol was low (between 9.0 and 12.5 vol %), helping us to understand the low extraction of tannins even after extended maceration. However, in a previous study carried out on Marquette grapes using two concentrations of ethanol (15 vol % and 50 vol %), none of those concentrations improved the concentration of tannins in the model wine solutions, suggesting that the structure and composition of cells negatively impact the extraction of tannins in those grape cultivars [36]. It has been previously reported that cold-hardy grape cultivars such as Marquette and Frontenac are rich in proteins and polysaccharides [37,38,39] which can interact with tannins, leading to a reduction in their content in wines. Polysaccharides and proteins are extracted similarly to tannins after a few days of alcoholic fermentation due to the disruption of cells. In addition, Garrido-Bañuelos et al. [40] observed a continuous depectination of grape solids during extended maceration, which could solubilize and impact their interactions with tannins. From these findings, the hypothesis that was formulated is that EM increases the extraction of proteins and polysaccharides from grapes, negatively impacting the tannin concentration in those wines at pressing. However, further work is necessary on the quantification of polysaccharides and proteins as well as on the methods used to quantify tannins, in order to fully understand the reasons behind those low tannin concentrations in wines.

### 3.3. Color Parameters, Anthocyanin Concentration, SPP

Interspecific grape cultivars are rich in anthocyanins, with sometimes 10 times more than what can be found in a *Vitis vinifera* grape cultivar, with both mono- and diglucoside forms, that does not have the same reaction rate, thus differently impacting the color stability [41].

**Figure 2:**
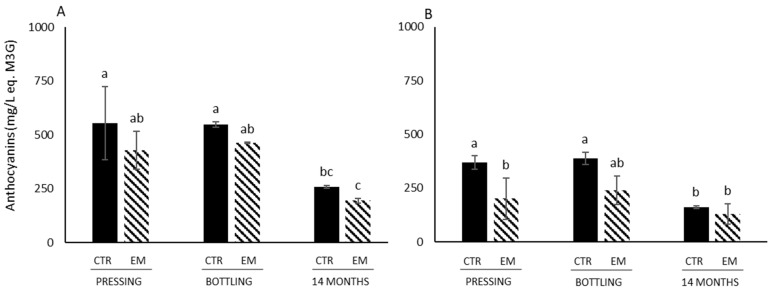
Total anthocyanin content (mg/L equivalent Malvidin-3-glucoside) in EM wines. (**A**) refers to wines made with Marquette cultivar, and (**B**) refers to wines made with Petite Pearl cultivar. Different letters indicate statistically significant differences (*p* < 0.05) by two-way ANOVA (time point versus treatment) followed by Tukey’s test.

EM did not have a statistically significant effect on hue, color intensity, or L* a* b* values in wines made from Marquette (Table 3). However, hue significantly increased from pressing to 14 months of aging for both the control and EM wines, and color intensity significantly decreased over the same period. No statistically significant difference was observed in L* values between these time points (Table 3). EM only had a statistically significant effect on the hue at bottling, on the color intensity at pressing, on a* at pressing, and on b* both at pressing and after 14 months of storage in wines made from Petite Pearl (Table 3). In Petite Pearl wines, the concentration of anthocyanins (Figure 2) was lower than in Marquette wines, which could explain the marked changes in color parameters. At the same time, hue was statistically increasing for both control and EM wines between pressing and 14 months of storage, and color intensity was decreasing only for control wines for the same period (Table 3).

Total anthocyanin concentrations in Marquette wines varied from 193.5 mg/L M3G Eq. (after 14 months EM) to 573.9 mg/L M3G Eq. (pressing control), and from 130.1 mg/L M3G Eq. (after 14 months EM) to 388.5 mg/L M3G Eq. (bottling control) in Petite Pearl wines (Figure 2).

There was a significant decrease in anthocyanin concentrations between bottling and 14 months of aging in Marquette wines for both treatments (control and EM). Similarly, there was a statistical decrease in anthocyanin content between bottling and 14 months of aging for Petite Pearl wines only for the control (Figure 2). These results were in agreement with previous studies observing that anthocyanins are extracted and more soluble in polar solvent (aqueous solution of juice) and that their extraction and concentration decrease from 5 days after crushing [42]. No statistical differences were determined between pressing and bottling for all conditions. Surprisingly, EM did not statistically impact the anthocyanin concentration for both Marquette and Petite Pearl wines (Figure 2). In previous studies evaluating the impact of EM on anthocyanins concentration in Cabernet sauvignon and Merlot wines, it was observed that the anthocyanin concentration decreased after EM compared to control wines likely due to the formation of small and large polymeric pigments and the adsorption of anthocyanins to cell wall material [14,42]. Yeast mannoproteins are released during and after alcoholic fermentation mainly through yeast autolysis that can take up to 4 weeks of maceration. Grape polysaccharides rich in arabinose, galactose and rhamnose are extracted throughout fermentation and maceration and the length of maceration is positively correlated with the concentration of polysaccharides rich in arabinose and galactose (PRAGs) [17]. Anthocyanins can adsorb to cell wall material within the first 60 min of contact, but can also desorb quickly. This can lead to a decrease in the anthocyanin concentration during the winemaking process [43,44]. Anthocyanin diglucosides that are the main form of anthocyanins found in cold-hardy grape varieties interact at different rates and with different strengths with polysaccharides. However, as previously observed [37], anthocyanin diglucosides interact less with flesh tissue than anthocyanin monoglucoside, suggesting that the decrease in the anthocyanin concentration after EM was due to both the type of anthocyanin and the cell wall material.

**Figure 3:**
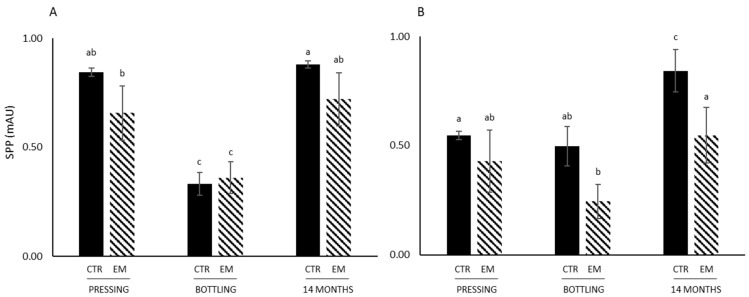
Small Polymeric Pigments (SPPs) in EM wines. (**A**) refers to wines made with Marquette cultivar, and (**B**) refers to wines made with Petite Pearl cultivar. Different letters indicate statistically significant differences (*p* < 0.05) by two-way ANOVA (time point versus treatment) followed by Tukey’s test.

Small Polymeric Pigments (SPPs) in Petite Pearl wines were stable from pressing to bottling and then statistically significantly increased after 14 months of aging (Figure 3). In Marquette wines, SPP were the same level at pressing and after 14 months of aging. EM did not statistically impact the level of SPP in Marquette wines at any time point (Figure 3), which was likely related to the lack of impact of EM on the concentration of anthocyanins. However, in Petite Pearl wines, the level of SPP was statistically lower in EM wines, at bottling and after 14 months of aging (Figure 3). These results were in agreement with previous work observing a decrease in SPP level after 30 and 250 days in Cabernet sauvignon wines that underwent EM [42]. SPPs were less formed after EM most likely due to the low anthocyanin concentration in wines and low tannin concentrations. These results are grape-cultivar-dependent, and show that the anthocyanin/tannin ratio is important to understand the formation of polymeric pigments in wines, as previously suggested [6,45].

## 4. Conclusions

This study evaluated the effect of two weeks’ EM on the phenolics concentrations (tannins and anthocyanins) and color parameters of wines made from interspecific grape cultivars. Although it was expected to observe a higher concentration of tannins in those wines after EM, the opposite was observed for both cultivars at pressing. The concentration of tannins was lower than 200 mg/L and the concentration of anthocyanins was high (around 550 mg/L), which potentially led to no impact of EM on color and tannin concentrations of Marquette wines. Astringency is a mouthfeel resulting from the interactions between tannins and salivary proteins and precipitation of the formed complexes. However, as a result of the lack of impact of EM on tannin and anthocyanin concentrations, and as the concentration of tannins was low, it was unexpected to observe a change in the sensory profile, especially astringency, of these wines. Also, as these grape cultivars are rich in other macromolecules, including pectins and proteins, that could be extracted during EM and could interact with phenolic compounds, this suggests that this technique might not be the most suitable to improve tannin extraction and retention in wines made from these interspecific grape cultivars. Further study on the same grape cultivar during another growing season and including a chemical analysis of other macromolecules is necessary prior to draw further conclusions on the effect of this technique on wines made from interspecific grape cultivars.

## Figures and Tables

**Table 1:** Physical and chemical parameters of Marquette and Petite Pearl cultivar wines at crushing.

	Marquette	Petite Pearl	*p*-Value
Brix	22.43 ± 1.29 ^‡^	19.00 ± 0.10	0.021
pH	2.99 ± 0.08	3.15 ± 0.02	0.961
Titratable acidity (g/L)	13.55 ± 1.15 ^‡^	8.21 ± 0.30	0.005
Tartaric acid (g/L)	8.89 ± 0.52 ^‡^	7.10 ± 0.10	0.012
Malic acid (g/L)	8.06 ± 1.40 ^‡^	2.53 ± 0.12	0.010
Citric acid (mg/L)	0.50 ± 0.06 ^‡^	0.34 ± 0.01	0.019
Hue	0.93 ± 0.52	0.49 ± 0.07	0.139
Color intensity (AU)	4.42 ± 1.02	7.25 ± 3.03	0.878
L*	45.47 ± 6.39	30.77 ± 9.78	0.053
a*	52.06 ± 11.36	53.60 ± 6.50	0.572
b*	52.95 ± 2.57 ^‡^	40.15 ± 6.43	0.029
Total phenolics (mg/L)	308.3 ± 111.9	342.30 ± 16.30	0.673
Tannin (mg/L)	16.33 ± 4.85	21.51 ± 5.91	0.846
Anthocyanins (mg/L)	542.2 ± 11.4^‡^	109.90 ± 47.00	0.001
SPP	1.10 ± 0.50	0.33 ± 0.14	0.052

Values are presented as mean ± standard deviation (*n* = 3). Superscript double dagger ^‡^ indicates statistical differences, using Student’s *t*-test (α: 0.05), between the two cultivars.

**Table 2:** Organic acid and alcohol concentrations in wines at pressing, bottling and after 14 months of aging.

		Marquette		Petite Pearl	
		Control	EM	Control	EM
pH	Pressing	3.20 ± 0.03 ^a^	3.30 ± 0.11 ^a^	3.22 ± 0.04 ^a^	3.33 ± 0.07 ^a^
	Bottling	3.26 ± 0.04 ^a^	3.39 ± 0.06 ^a^	3.18 ± 0.04 ^a^	3.25 ± 0.03 ^a^
	14 months	3.20 ± 0.01 ^a^	3.28 ± 0.14 ^a^	3.18 ± 0.07 ^a^	3.23 ± 0.09 ^a^
Titratable acidity (g/L)	Pressing	12.58 ± 0.74 ^a^	10.66 ± 1.41 ^ab^	9.57 ± 0.98 ^a^	9.21 ± 0.68 ^ab^
	Bottling	8.74 ± 0.24 ^bc^	8.55 ± 0.15 ^bc^	8.07 ± 0.28 ^ab^	7.80 ± 0.36 ^ab^
	14 months	7.91 ± 0.27 ^c^	9.20 ± 1.66 ^bc^	7.75 ± 1.13 ^ab^	7.35 ± 0.65 ^b^
Tartaric acid (g/L)	Pressing	5.87 ± 0.15 ^a^	5.05 ± 0.42 ^b^	6.36 ± 0.56 ^a^	5.05 ± 0.85 ^ab^
	Bottling	3.47 ± 0.19 ^d^	3.11 ± 0.30 ^d^	3.90 ± 0.44 ^b^	3.61 ± 0.37 ^b^
	14 months	4.22 ± 0.01 ^c^	4.48 ± 0.25 ^bc^	4.93 ± 0.30 ^ab^	4.32 ± 0.91 ^b^
Malic acid (g/L)	Pressing	5.02 ± 0.07 ^a^	0.02 ± 0.01 ^b^	0.85 ± 0.02 ^a^	0.02 ± 0.01 ^b^
	Bottling	0.02 ± 0.01 ^b^	0.02 ± 0.01 ^b^	0.02 ± 0.01 ^b^	0.02 ± 0.01 ^b^
	14 months	nd	nd	nd	nd
Lactic acid (g/L)	Pressing	0.08 ± 0.01 ^b^	1.37 ± 0.23 ^a^	0.28 ± 0.07 ^b^	0.64 ± 0.04 ^a^
	Bottling	1.38 ± 0.01 ^a^	1.37 ± 0.21 ^a^	0.60 ± 0.07 ^a^	0.65 ± 0.04 ^a^
	14 months	1.63 ± 0.01 ^a^	1.57 ± 0.34 ^a^	0.73 ± 0.15 ^a^	0.80 ± 0.05 ^a^
Acetic acid (g/L)	Pressing	0.32 ± 0.02 ^e^	0.45 ± 0.00 ^b^	0.29 ± 0.01 ^b^	0.42 ± 0.01 ^a^
	Bottling	0.37 ± 0.01 ^d^	0.47 ± 0.01 ^b^	0.38 ± 0.01 ^ab^	0.42 ± 0.02 ^ab^
	14 months	0.41 ± 0.00 ^c^	0.54 ± 0.02 ^a^	0.29 ± 0.12 ^b^	0.50 ± 0.06 ^a^
Ethanol(vol %)	Pressing	11.38 ± 0.07 ^a^	11.53 ± 0.61 ^a^	9.42 ± 0.48 ^a^	8.62 ± 0.96 ^a^
	Bottling	11.99 ± 0.20 ^a^	12.04 ± 0.89 ^a^	9.55 ± 0.37 ^a^	8.73 ± 0.96 ^a^
	14 months	11.97 ± 0.29 ^a^	11.95 ± 0.97 ^a^	9.60 ± 0.26 ^a^	9.39 ± 0.85 ^a^

Values are presented as mean ± standard deviation (*n* = 3). Different superscript letters indicate statistically significant differences (*p* < 0.05) by two-way ANOVA (time point across treatment) for each individual cultivars, followed by Tukey’s test. Two-way ANOVA *p*-values are displayed in the Supplementary Table S1.

**Table 3:** Color parameters of red wines at pressing and bottling and after 14 months of aging.

		Marquette		Petite Pearl	
		Control	EM	Control	EM
Hue	Pressing	0.39 ± 0.01 ^c^	0.44 ± 0.04 ^bc^	0.39 ± 0.01 ^d^	0.50 ± 0.04 ^cd^
	Bottling	0.45 ± 0.01 ^bc^	0.53 ± 0.04 ^b^	0.45 ± 0.02 ^d^	0.57 ± 0.01 ^bc^
	14 months	0.75 ± 0.01 ^a^	0.77 ± 0.10 ^a^	0.62 ± 0.03 ^ab^	0.73 ± 0.09 ^a^
Color Intensity	Pressing	16.40 ± 0.96 ^a^	13.53 ± 3.34 ^ab^	15.42 ± 1.51 ^a^	6.66 ± 2.04 ^b^
	Bottling	10.53 ± 0.89 ^bc^	8.13 ± 1.93 ^c^	10.48 ± 0.95 ^b^	5.43 ± 2.04 ^b^
	14 months	10.29 ± 0.07 ^bc^	8.10 ± 1.37 ^c^	10.17 ± 0.95 ^b^	6.20 ± 1.03 ^b^
L*	Pressing	16.30 ± 1.45 ^b^	19.37 ± 4.11 ^ab^	13.70 ± 1.67 ^b^	28.93 ± 7.53 ^ab^
	Bottling	23.30 ± 1.97 ^ab^	26.80 ± 5.31 ^a^	18.97 ± 0.29 ^ab^	31.00 ± 11.17 ^a^
	14 months	15.00 ± 0.10 ^b^	21.30 ± 3.47 ^ab^	15.70 ± 2.82 ^ab^	26.67 ± 4.48 ^ab^
a*	Pressing	45.35 ± 1.69 ^bc^	48.08 ± 4.46 ^abc^	42.48 ± 1.88 ^b^	54.76 ± 4.79 ^a^
	Bottling	52.28 ± 2.19 ^ab^	54.34 ± 4.07 ^a^	47.63 ± 0.33 ^ab^	52.71 ± 7.24 ^ab^
	14 months	41.82 ± 0.02 ^c^	48.54 ± 2.97 ^abc^	44.01 ± 3.54 ^ab^	52.14 ± 2.30 ^ab^
b*	Pressing	28.06 ± 2.53 ^b^	32.50 ± 6.68 ^ab^	23.53 ± 2.75 ^c^	32.71 ± 4.55 ^ab^
	Bottling	38.99 ± 2.78 ^a^	39.51 ± 2.03 ^a^	31.53 ± 0.29 ^abc^	25.02 ± 2.32 ^bc^
	14 months	25.60 ± 0.21 ^b^	34.26 ± 4.09 ^ab^	26.41 ± 4.29 ^bc^	35.26 ± 0.56 ^a^

Values are presented as mean ± standard deviation (*n* = 3). Different superscript letters indicate statistically significant differences (*p* < 0.05) by two-way ANOVA (time point across treatment) for each individual cultivar, followed by Tukey’s test. Two-way ANOVA *p*-values are displayed in the Supplementary Table S1.

## Data Availability

All data underlying this study are included in the article and its Supplementary Materials.

## Supplementary Materials

The following supporting information can be downloaded at: https://www.mdpi.com/article/10.3390/foods14071187/s1, Table S1. Two-way ANOVA *p*-values of chemical parameters of Marquette and Petite Pearl wines. Figure S1. Evolution of the temperature and the degree Brix during alcoholic fermentation of Marquette (A) and Petite Pearl (B) grapes.

## Abbreviations

The following abbreviations are used in this manuscript:

SPP	Small Polymeric Pigment
mDP	mean Degree of Polymerization
EM	extended maceration
IRP	iron-reactive phenolic
TA	titratable acidity
