# Supplementary material for: How Does Extended Maceration Affect Tannin and Color of Red Wines from Cold-Hardy Grape Cultivars?
Source: Foods. 2025 Mar 28;14(7):1187. doi: 10.3390/foods14071187 (PMC11988643; doi:10.3390/foods14071187)

How Does Extended Maceration Affect Tannin and Color of Red Wines from Cold-Hardy Grape Cultivars?

Aude A. Watrelot, and Nicolas Delchier

Table S1: Two-way ANOVA p-values of chemical parameters of Marquette and Petite Pearl wines.

		p-value	
		Marquette	Petite Pearl
pH	Treatment	0.3530	0.0163
	Time point	0.6395	0.1224
	Treatment × Time point	0.0286	0.7152
Titratable acidity	Treatment	0.5540	0.3480
	Time point	0.0001	0.0026
	Treatment × Time point	0.0398	0.9893
Tartaric acid	Treatment	0.0246	0.0266
	Time point	<0.0001	0.0005
	Treatment × Time point	0.0097	0.3736
Malic acid	Treatment	<0.0001	<0.0001
	Time point	<0.0001	<0.0001
	Treatment × Time point	<0.0001	<0.0001
Lactic acid	Treatment	0.0006	0.0012
	Time point	<0.0001	<0.0001
	Treatment × Time point	<0.0001	0.0080
Acetic acid	Treatment	<0.0001	0.0001
	Time point	<0.0001	0.2896
	Treatment × Time point	0.0545	0.0364
Citric acid	Treatment	<0.0001	0.0234
	Time point	0.0003	<0.0001
	Treatment × Time point	<0.0001	<0.0001
Succinic acid	Treatment	0.5611	0.0019
	Time point	<0.0001	<0.0001
	Treatment × Time point	0.6938	0.0122
Ethanol	Treatment	0.8556	0.0948
	Time point	0.2531	0.5062
	Treatment × Time point	0.9706	0.7043
Glycerol	Treatment	0.5365	0.6618
	Time point	<0.0001	<0.0001
	Treatment × Time point	0.7385	0.9785
Hue	Treatment	0.0534	<0.0001
	Time point	<0.0001	<0.0001
	Treatment × Time point	0.4597	0.9752
Color Intensity	Treatment	0.0110	<0.0001
	Time point	0.0001	0.0271
	Treatment × Time point	0.9419	0.0722
L*	Treatment	0.0159	0.0007
	Time point	0.0034	0.4765
	Treatment × Time point	0.6545	0.8142
a*	Treatment	0.0179	0.0007
	Time point	0.0011	0.6547
	Treatment × Time point	0.3717	0.3314
b*	Treatment	0.0215	0.0175
	Time point	0.0011	0.2436
	Treatment × Time point	0.1965	0.0008

Figure S1. Evolution of the temperature and the degree Brix during alcoholic fermentation of Marquette (A) and Petite Pearl (B) grapes.

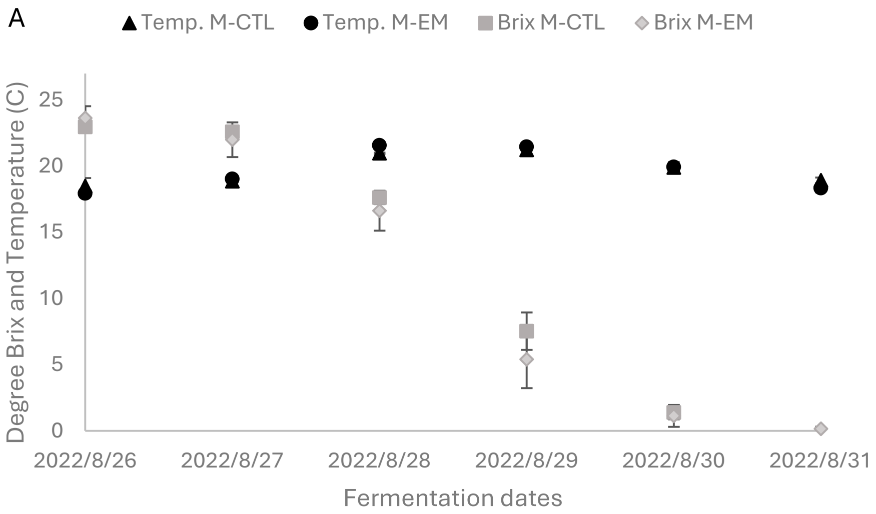

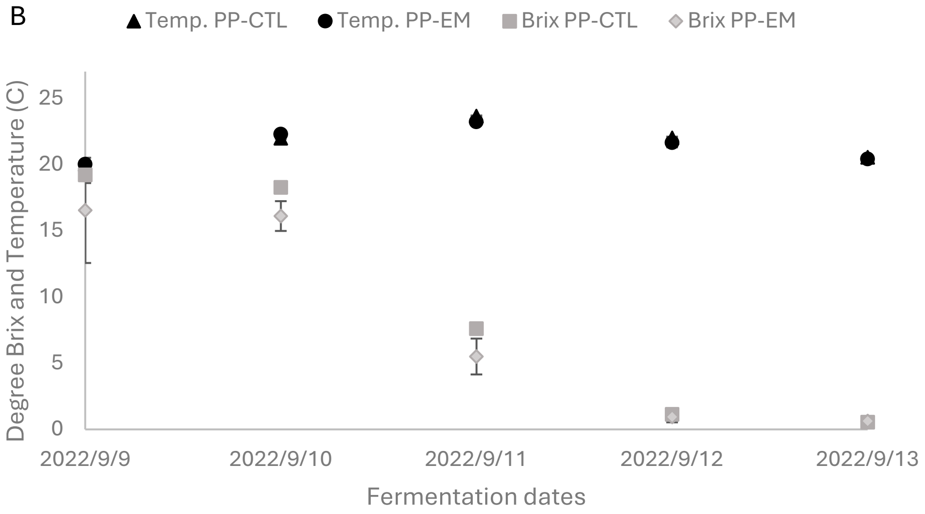

Supplement: Supplementary file 1 [file foods-14-01187-s001.zip › foods-3529479-supplementary.pdf]
